# Photon-counting CT of degenerative changes and rupture of silicone breast implants: a pilot study

**DOI:** 10.1186/s41747-024-00434-4

**Published:** 2024-03-14

**Authors:** Claudia Neubauer, Oliver Gebler, Caroline Wilpert, Maxim Scherwitz, Niklas Efinger, Fabian Bamberg, Marisa Windfuhr-Blum, Jakob Neubauer

**Affiliations:** Department of Radiology, Medical Center – University of Freiburg, Faculty of Medicine, University of Freiburg, Freiburg, Germany

**Keywords:** Breast implants, Magnetic resonance imaging, Photon-counting computed tomography, Sensitivity and specificity, Silicone gels

## Abstract

**Background:**

Accurate assessment of breast implants is important for appropriate clinical management. We evaluated silicone properties and diagnostic accuracy for characterizing silicone implants and detecting degenerative changes including rupture in photon-counting computed tomography (PCCT).

**Methods:**

Over 16 months, we prospectively included patients with silicone implants and available breast magnetic resonance imaging (MRI) who received thoracic PCCT performed in prone position. Consensus reading of all available imaging studies including MRI served as reference standard. Two readers evaluated all implants in PCCT reconstructions for degenerative changes. In a subgroup of implants, mean density of silicone, adjacent muscle, and fat were measured on PCCT reconstructions. Contrast-to-noise ratios (CNRs) were calculated for implant-to-muscle and implant-to-fat.

**Results:**

Among 21 subjects, aged 60 ± 13.1 years (mean ± standard deviation) with 29 implants PCCT showed the following: high accuracy for linguine sign, intraimplant fluid (all > 0.99), peri-implant silicone (0.95), keyhole sign (0.90), and folds of the membrane (0.81); high specificity for linguine sign, intraimplant fluid, keyhole sign, folds of the membrane (all > 0.99), and peri-implant silicone (0.98); and high sensitivity for linguine sign and intraimplant fluid (all > 0.99). In a subgroup of 12 implants, the highest CNR for implant-to-muscle was observed on virtual unenhanced reconstructions (20.9) and iodine maps (22.9), for implant-to-fat on iodine maps (27.7) and monoenergetic reconstructions (31.8).

**Conclusions:**

Our findings demonstrate that silicone breast implants exhibit distinct contrast properties at PCCT, which may provide incremental information for detection of degenerative changes and rupture of implants.

**Relevance statement:**

Thoracic photon-counting computed tomography is a promising modality for the diagnostic assessment of silicone breast implants.

**Key points:**

• Thoracic photon-counting computed tomography demonstrates unique contrast properties of silicone breast implants.

• Iodine map reconstructions reveal strong contrast-to-noise ratios for implant-to-muscle and implant-to-fat.

• Thoracic photon-counting computed tomography shows high diagnostic accuracy in detecting implant degeneration and rupture.

**Trial registration:**

German Clinical Trials Register number DRKS00028997, date of registration 2022–08-08, retrospectively registered.

**Graphical Abstract:**

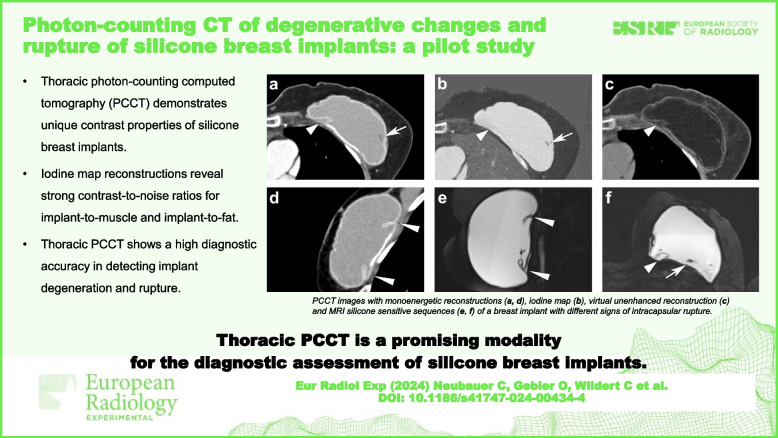

## Background

Breast reconstruction with silicone implants is a widely performed cosmetic procedure for breast augmentation due to aesthetic purposes, following congenital malformations or after mastectomy in breast cancer patients. Depending on the gel cohesivity, surface structure, implant age, and location, silicone implants show a different range of degradation over time including microscopic silicone gel bleed through otherwise intact implant membranes [1] and intracapsular or extracapsular rupture [2–4]. All instances may lead to further complications [1, 5, 6] and the need of surgical removal of the implant or even the surrounding tissue and lymph nodes.

Intracapsular implant ruptures within the fibrotic capsule occur more frequently than extracapsular ruptures [2]. They can be asymptomatic and difficult or even impossible to detect at clinical examination, mammography, or ultrasound [7]. Breast MRI with silicone selective sequences represents the most sensitive procedure to detect implant ruptures [7–10]. In MRI scans, the major signs of implant rupture include peri-implant silicone collections and the linguine sign; in uncollapsed or minimally collapsed intracapsular ruptures, the subcapsular line, keyhole, noose, or teardrop signs may become apparent [4, 7, 9, 11]. With mixture of saline and silicone, the unspecific salad oil sign or droplet sign might be included. It is also possible for calcifications to form on the capsule over time, independently of a possible rupture [7].

Implant ruptures may also incidentally be seen in imaging including computed tomography (CT) examinations [12, 13]. Despite the visualization of implants and their complications in routine CT [14], a limitation for single-energy CT as a diagnostic tool for ruptured implants is the similar radiodensity of silicone and soft breast tissue. In contrast, dual-energy CT performs similar to MRI for the detection of silicone implant ruptures [12, 13]. Also, dedicated photon-counting breast CT provides promising results for the evaluation of implant integrity or extensive capsular fibrosis beside other non-implant-related features such as breast density, soft tissue lesions, or micro- and macrocalcifications in the tissue surrounding the implants [15].

Whole-body photon-counting CT (PCCT) is a new promising CT technology [16]. So far, to our knowledge, no study regarding the assessment of silicone breast implants exists for thoracic PCCT.

Therefore, the purpose of our present study was to evaluate the contrast properties of silicone breast implants in thoracic PCCT and to identify the reconstruction technique that yields the best image contrast for silicone. In addition, we evaluated the diagnostic accuracy of PCCT in detecting degenerative changes and rupture of silicone breast implants as accurate detection and diagnosis of these complications are important for appropriate clinical management.

## Methods

### Study design and population

Our prospective cohort study was performed at the University Medical Center Freiburg, Germany, over 16 months from January 2022 to May 2023. All female patients with silicone breast implants who had a clinical indication for thoracic CT imaging and available breast MRI studies were included. Clinical indications for thoracic CT were thoracic and abdominal staging examinations under chemotherapy in metastatic breast cancer (11 patients), ipsilateral recurrence (3 patients) or contralateral carcinoma after breast carcinoma (2 patients), initial staging after newly diagnosed breast cancer (1 patient), restaging after bilateral breast carcinoma (1 patient), restaging after breast cancer due to unclear liver lesions (1 patient) or thoracic CT follow-up of pulmonary nodules (1 patient), and thoracic CT as exclusion of pulmonary nodules after breast cancer (1 patient). Written informed consent was obtained from all study participants. Our study was conducted in accordance with the Declaration of Helsinki and approved by the local ethics committee (number 21–1717).

### Imaging protocols

#### PCCT

**Fig. 1:**
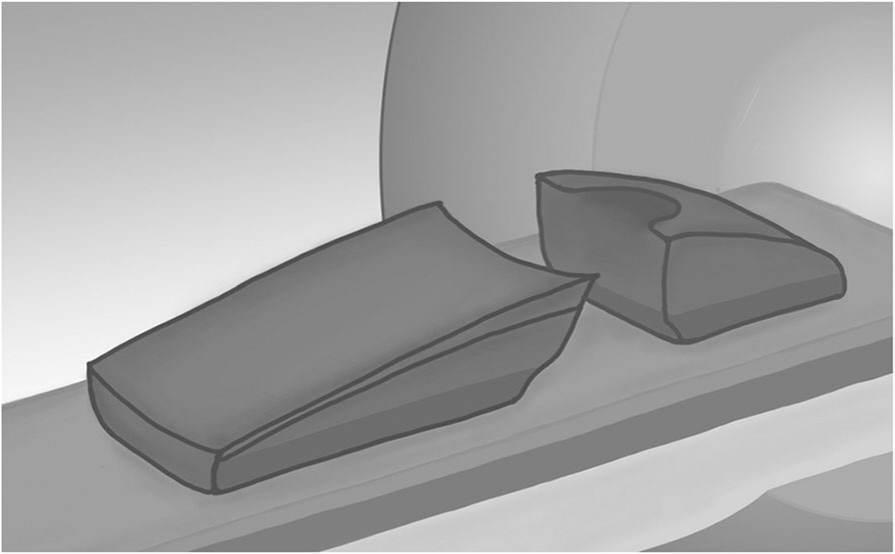
Sketch of the positioning aid for patients in prone position with both breasts hanging freely in photon-counting computed tomography like in breast magnetic resonance imaging

All participants underwent a PCCT examination (NAEOTOM Alpha, Siemens Healthineers, Erlangen, Germany) in prone position. Both breasts were hanging freely between positioning pillows similar to those used in MRI examinations of the breast (Fig. 1). Examinations were performed using a helical acquisition and a fixed delay of 85 s after bodyweight-adapted bolus injection of iodinated contrast agent (iopromide 370 mg/mL, Bayer Healthcare, Leverkusen, Germany) and saline chaser with a flow of 3 mL/s via a 20-gauge catheter.

Tube voltage was set to 120 kVp with a constant quality reference of 142 mAs and automatic dose optimization. Therefore, the radiation dose indicators varied based on the body size and constitution of patients and additionally depended on whether solely a thoracic or a thoracoabdominal examination had to be performed. Additional reconstructions covering both breasts, the anterior thoracic wall, and the axillary region, including a transversal monoenergetic 65 keV, iodine map, and virtual unenhanced reconstruction, were performed and used for evaluation in this study. These reconstructions implied a field of view of 34 cm, a matrix of 1024 × 1024 pixels, a slice thickness of 2 mm, and a slice increment of 2 mm, kernel Br40, and iterative reconstruction strength 3. Information left on those pseudonymized reconstructions included slice number, slice thickness and interval, keV, mAs, level and width of reconstruction, and reconstruction name as those parameters were implemented in all prone-positioned PCCT examinations and reconstructions. All reconstructions were easily identifiable by image appearance anyway; therefore, hiding those parameters would not have influenced the outcome, while patient data, investigation date, and time were hidden.

#### MRI

All participants underwent standard multiparametric 1.5-T or 3-T breast MRI with application of contrast agent. At our hospital, for 9 of 21 patients, we used an 18-channel breast coil and 0.1 mmol/kg of contrast agent (gadoteridol, ProHance, Bracco, Konstanz, Germany). As breast MRIs were performed externally in 12 of 21 patients, protocols varied regarding general and silicone-sensitive sequences for implant assessment.

### Reader study

#### Characterization of silicone breast implants

In the first 13 patients recruited over the initial period of 9 months, the integrity of the breast implants was assessed by two experienced breast radiologists (with 11 and 25 years of experience) in a consensus reading of all modalities including MRI. In order to prevent the values from being falsified by degeneratively altered implants, only implants that showed no signs of rupture in the MRI were used for the quantitative analysis (12 implants), while 1 implant had to be excluded due to silicone collections outside the implant membrane and presence of the linguine sign. Two readers (one subspeciality trained breast radiologists with 10 years of experience and one medical student with 1 year experience handling PCCT data in the Picture Archiving and Communication System), who were blinded for all patient data, independently evaluated the monoenergetic (65 keV) reconstruction, the iodine map, and the virtual unenhanced reconstruction of pseudonymized PCCT images. Mean density and standard deviation of intact silicone implants, adjacent muscle, and adjacent fat were measured in Hounsfield units using region-of-interest analysis. The examinations were evaluated with a viewing software (DeepUnity Diagnost, Dedalus, Bonn, Germany) under standard diagnostic conditions.

#### Analysis of degenerative changes of silicone breast implants

Two experienced breast radiologists (with 5 and 10 years of experience) independently evaluated breast implants of all included patients on pseudonymized PCCT images for the presence of folds of the membrane, peri-implant fluid collections, peri-implant silicone collections, intraimplant fluid, keyhole and linguine sign, and the presence of capsular calcifications. Transversal pseudonymized monoenergetic (65 keV) reconstructions, a transversal iodine map and transversal virtual unenhanced reconstructions covering both breasts were presented as well as an additional coronal monoenergetic reconstruction for orientation purposes. Assessment quality of implants and breast tissue was evaluated using a Likert scale (1 = very good, 2 = good, 3 = moderate, 4 = bad).

As reference standard, a consensus reading by two radiologists (11 and 25 years of experience in breast imaging) was performed of all available imaging examinations including MRI, digital mammography, and/or tomosynthesis with MRI being predominantly used to evaluate all signs of degeneration. As MRI and even mammography and tomosynthesis cannot serve as a reliable reference standard for capsular calcifications, we excluded capsular calcifications from the diagnostic accuracy assessment and only calculated inter-rater reliability.

### Statistics

Categorical parameters were represented using both absolute and relative frequencies, while continuous parameters were depicted through median values and their corresponding interquartile range. CNR was calculated for implant-to-muscle and implant-to-fat ratio and compared between monoenergetic reconstructions and material decomposition (iodine map and virtual unenhanced reconstructions) using Friedman test, followed by post hoc Nemenyi test. Regarding diagnostic accuracy of PCCT, pooled measures with 95% confidence interval (CI), and for inter-rater reliability, Cohen’s κ was calculated, with κ values interpreted as follows: 0.00, no agreement; *κ* ≤ 0.20, slight agreement; 0.20 < *κ* ≤ 0.40, fair agreement; 0.40 < *κ* ≤ 0.60, moderate agreement; 0.60 < *κ* ≤ 0.80, substantial agreement; and 0.80 < *κ* ≤ 1.00, almost perfect agreement [17]. We considered a *p*-value < 0.05 to indicate statistical significance. All statistical analyses were performed using R version 4.3.0 (the R Foundation for Statistical Computing, Vienna, Austria).

## Results

### Implant properties

Altogether, 21 female patients with 29 silicone-containing implants could be included in this study. Patient’s age at implantation of prosthesis varied between 26 and 81 years (mean age of 47 years, unknown in 2 patients). Patient’s age at time of examination in the context of our study varied between 41 and 85 years (60 ± 13.1 years, mean ± standard deviation). The length of time since implantation varied between 2 and 24 years (mean value 10 years, unknown in 2 patients). Reasons for prosthesis implantation were *ablatio mammae* after ipsilateral breast cancer (14 implants), bilateral *ablatio mammae* due to bilateral breast cancer (4 patients with 8 implants), protective *ablatio mammae* with reconstruction after contralateral breast cancer (2 implants), breast reconstruction after contralateral breast cancer (3 implants), and breast augmentation (2 implants). Implants were located unilaterally on the left side in 8 cases, on the right side in 5 cases, and bilaterally in 8 cases; 19 implants were located subpectorally and 10 epipectorally. As most of the surgical implantations were performed in external hospitals, only in 12 patients with 17 implants details about the implants were identifiable. Most of those implants were textured or polyurethan foamed prosthesis.

### Characterization of implants at the reference standard

In the consensus reading of all modalities including MRI, signs of implant degeneration were found in 58.6% of implants (17/29) with collapsed intracapsular rupture in 6.9% (2/29) and signs of uncollapsed or minimally collapsed rupture in 51.7% (15/29), while 3.4% (1/29) only showed unspecific signs of rupture, and 37.9% of implants showed no signs of degeneration at all or only radial folds (11/29). The linguine sign was identified in 3.4% (1/29), the keyhole sign in 58.6% (17/29), peri-implant silicone collections in 6.9% (2/29), peri-implant nonsilicone fluid collections in 24.1% (7/29), intraimplant fluid in 13.8% (4/29), and folds of the membrane in 75.9% (22/29).

### Characterization of silicone implants

**Fig. 2:**
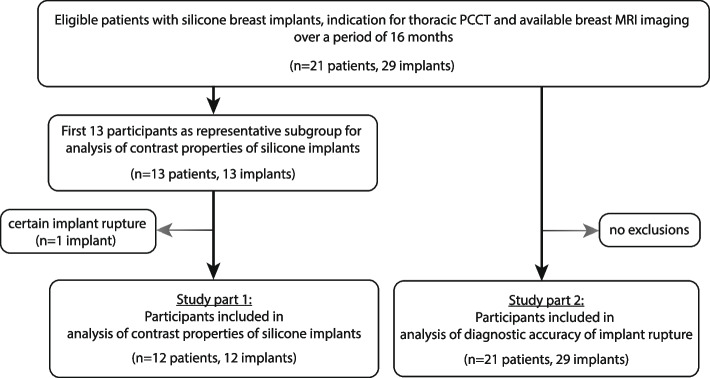
Flowchart showing patient recruitment for study part 1 and study part 2. *MRI* Magnetic resonance imaging, *PCCT* Photon-counting computed tomography

In the first part of our study, we were able to include a representative subpopulation of the first 12 acquired patients with 12 implants after exclusion of 1 patient with 1 implant showing signs of collapsed implant rupture (flowchart in Fig. 2).

**Fig. 3:**
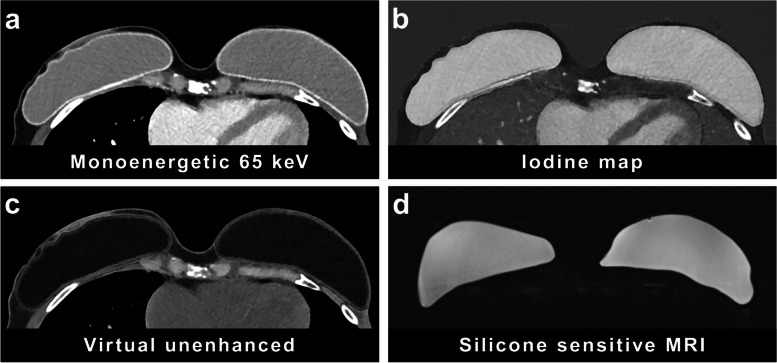
Photon-counting computed tomography images (**a**–**c**) and magnetic resonance imaging images with silicone-sensitive sequences (**d**) of two intact subpectoral breast implants on both sides. Axial monoenergetic 65-keV reconstruction (**a**) and material decomposition reconstructions (iodine map [**b**] and virtual unenhanced [**c**]) show distinct contrast properties of the implants with high density of the silicone in the iodine map with window level 40 HU and window width 400 HU (**b**)

The reconstruction technique with the highest CNR for implant-to-muscle was found to be the virtual unenhanced reconstruction (CNR of 17.51 for reader 1, 24.28 for reader 2) and the iodine map (CNR of 22.92 for reader 1, 22.95 for reader 2) (compare Table 1 with pooled values for both readers, Fig. 3). The monoenergetic reconstructions showed significant lower CNRs for implant-to-muscle (6.15 for reader 1, 7.04 for reader 2, in comparison to the iodine map with *p* < 0.001 for both readers and to the virtual unenhanced reconstruction, with *p* = 0.010 for reader 1 and 0.002 for reader 2). The highest CNR values for implant-to-fat were demonstrated in the iodine map (CNR of 28.02 for reader 1, 27.37 for reader 2) and regular monoenergetic reconstruction (CNR of 28.93 for reader 1, 34.64 for reader 2), while the virtual unenhanced reconstructions yielded significantly poorer CNRs (2.63 for reader 1, 3.16 for reader 2, in comparison to the iodine map with *p* < 0.001 for reader 1 and *p* = 0.006 for reader 2 and to the virtual unenhanced reconstruction with *p* < 0.001 for both readers) (compare Table 1 with pooled values for both readers, Fig. 3).

**Table 1:** Pooled CNR for implant-to-muscle and implant-to-fat for virtual unenhanced, iodine map, and monoenergetic (65 keV) reconstruction with *p*-values between all groups

	Virtual unenhanced	Iodine map	Regular monoenergetic	*p*-value for all groups
CNR implant-to-muscle	20.9	22.9	6.6	< 0.001
CNR implant-to-fat	2.9	27.7	31.8	< 0.001

*CNR* Contrast-to-noise ratio

When reader 1 and reader 2 were pooled, CNR for implant-to-muscle was observed to be significantly higher in the iodine map and the virtual unenhanced reconstruction compared to the monoenergetic reconstruction with *p*-values < 0.001 as was CNR for implant-to-fat in the iodine map and monoenergetic reconstruction compared to the virtual unenhanced reconstruction.

### Diagnostic accuracy for degenerative changes of implants including rupture

**Fig. 4:**
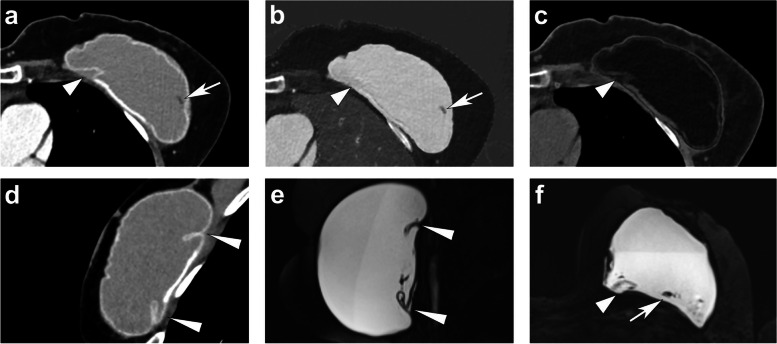
Photon-counting computed tomography images with monoenergetic reconstructions (**a** axial; **d** sagittal) and material decomposition reconstructions (axial iodine map [**b**] and virtual unenhanced [**c**]) as well as magnetic resonance imaging silicone-sensitive sequences (**e** sagittal; **f** axial) of a breast implant on the left side with intracapsular rupture. Peri-implant silicone collections (short thick arrowhead in **a**, **b**, **c**, **f**), keyhole sign (long thin arrowheads in **d** and **e**), and small intraimplant fluid collections (arrow in **a**, **b**, and **f**) can be detected on both modalities

In the second part of our study, we were able to include 21 patients with 29 implants. Of these examined implants, 58.6% (17/29) were degenerated with signs of collapsed or uncollapsed rupture (compare Fig. 4), and 3.4% (1/29) showed nonspecific signs for rupture, while 41.4% of implants (12/29) appeared to be intact according to the consensus reading of all examinations, predominantly of breast MRI, as mentioned above. We found that the diagnostic accuracy of PCCT was very high for detecting the linguine sign (> 0.99 [95% CI 0.94, > 0.99]), intraimplant fluid (> 0.99 [95% CI 0.94, > 0.99]), peri-implant silicone collections (0.95 [95% CI 0.86, 0.99]), the keyhole sign (0.90 [95% CI 0.79, 0.96]), and folds of the membrane (0.81 [95% CI 0.69, 0.90]) and good for peri-implant fluid collections (0.78 [95% CI 0.65, 0.87]) (Table 2).

**Table 2:** Diagnostic accuracy, sensitivity, specificity, and inter-rater reliability (Cohen κ) for signs of implant degeneration

	Accuracy (95% CI)	Sensitivity (95% CI)	Specificity (95% CI)	Inter-rater reliability
Folds	0.81 (0.69, 0.90)	0.75 (0.62, 0.88)	> 0.99 (0.70, > 0.99)	0.93
Keyhole sign	0.90 (0.79, 0.96)	0.82 (0.70, 0.95)	> 0.99 (0.86, > 0.99)	0.59
Fluid outside	0.78 (0.65, 0.87)	0.64 (0.39, 0.89)	0.82 (0.70, 0.93)	0.58
Fluid inside	> 0.99 (0.94, > 0.99)	> 0.99 (0.63, > 0.99)	> 0.99 (0.93, > 0.99)	> 0.99
Silicone outside	0.95 (0.86, 0.99)	0.50 (0.01, > 0.99)	0.98 (0.95, > 0.99)	0.65
Linguini sign	> 0.99 (0.94, > 0.99)	> 0.99 (0.16, > 0.99)	> 0.99 (0.94, > 0.99)	> 0.99

*CI* Confidence interval

Specificity was very high for the linguine sign, intraimplant fluid, the keyhole sign, folds of the membrane (each > 0.99), and peri-implant silicone collections (0.98 [95% CI 0.95, > 0.99]) (Table 2). Sensitivity was very high for the linguine sign and intraimplant fluid (each > 0.99) and high for the keyhole sign (0.82 [95% CI 0.70, 0.95]) and folds of the membrane (0.75 [95% CI 0.62, 0.88]).

Inter-rater reliability (Table 2) was nearly perfect for the linguine sign, intraimplant fluid, calcifications (each > 0.99), and for folds of the membrane (0.93) and substantial for peri-implant silicone collections (0.65). Inter-rater reliability was fair for peri-implant fluid collections (0.58) or the keyhole sign (0.59).

The average assessment of implants and breast tissue was rated good (with a medium of 2 = good on a Likert scale, interquartile range 1).

## Discussion

Our results demonstrate that silicone breast implants exhibit distinct contrast properties at PCCT with especially high CNRs for both implant-to-muscle and implant-to-fat in the iodine map. In addition, the virtual unenhanced reconstruction exhibited a high CNR for implant-to-muscle, and the monoenergetic reconstruction yielded a high CNR for implant-to-fat. Furthermore, PCCT proves to be highly accurate in detecting various signs of degenerative changes and rupture of silicone breast implants with a very high diagnostic accuracy for the linguine sign (> 0.99 [95% CI 0.94, > 0.99]), intraimplant fluid (> 0.99 [95% CI 0.94, > 0.99]), peri-implant silicone collections (0.95 [95% CI 0.86, 0.99]), and a high diagnostic accuracy for the keyhole sign (0.90 [95% CI 0.79, 0.96]) and folds of the membrane (0.81 [95% CI 0.69, 0.90]).

Silicone breast implants are foreign structures in the body and are frequently accompanied by several problems including not only difficulties in breast cancer screening and diagnosis [18] but also capsular contracture or implant rupture [1, 6]. The first-line diagnostic tools in breast imaging, mammography, tomosynthesis, and ultrasound reach their limits especially in the assessment of implant integrity in case of clinically silent intracapsular ruptures [7]. MRI in contrast shows the highest sensitivity for detection of intra- and extracapsular implant rupture [8, 10, 19]. For sole silicone implant evaluation, special silicone-sensitive sequences with water saturation and additional fat saturation are available and can be performed in axial and sagittal orientation without the need of contrast agent administration [7]. However, MRI is expensive, time-consuming, not available everywhere, and accompanied by contraindications.

So far, among the alternative evolving and promising methods for assessment of silicone breast implants and their complications, dual-energy CT [1, 12, 13, 20, 21] and dedicated breast CT [15, 22] can be found. Especially, thoracic CT scans could be of interest regarding their capability in assessing breast implant integrity, as patients with silicone implants might repeatedly have several reasons to receive thoracic CT imaging such as staging and control of side effects of therapy aside from completely different independent diseases needing thoracic/body CT imaging. With each thoracic CT scan, both breasts are displayed simultaneously. For thoracic CT imaging, PCCT is an emerging technology, where x-ray photons are directly converted into electrical signals in special detectors. Thus, advantages in image quality can be achieved including a higher spatial resolution, improved iodine signal, artifact reduction, and multienergy imaging accompanied by radiation dose reduction [16].

We showed that in thoracic PCCT imaging in prone position, silicone breast implants can be evaluated for degenerative changes and rupture with high accuracy especially as silicone shows distinct contrast properties in PCCT reconstructions. Signs of collapsed implant rupture were identified with a high diagnostic accuracy such as the linguine sign and peri-implant silicone collections. Also signs of uncollapsed implant rupture such as the keyhole sign show a high diagnostic accuracy. The very high specificity for the keyhole sign could be a result of the high resolution of PCCT, while the slightly reduced sensitivity might be due to the frequently not clearly identifiable content of the very small “keyholes.” Appearance of intraimplant fluid (diagnostic accuracy > 0.99) belongs to the unspecific signs of implant rupture in case of sole occurrence but might be a hint of implant rupture in combination with other signs. Similarly, it is the case with capsular calcifications, which might appear even without implant rupture. Still, they could be identified in PCCT with a high inter-rater reliability (> 0.99). Folds of the membrane and peri-implant fluid collections presented with a lower diagnostic accuracy (0.81, and 0.78, respectively). Peri-implant fluid collections might have been missed or misinterpreted due to the lower and more ambiguous contrast of soft tissues in CT compared to MRI as reference standard. The difference in assessment of folds of the membrane might not only be due to degenerative changes but also follow slight variations of positioning in PCCT and MRI. As both signs are not specific for implant rupture, but are regularly associated with physiological changes of implants, missing those signs in some cases might not be of major relevance for ruling out implant ruptures. Therefore, PCCT imaging performed for other indications appears to be a promising tool to analyze silicone implant integrity. Hence, in case of thoracic PCCT imaging, additional breast MRI for implant assessment might be omitted in future.

Moreover, when contrast agent is administered, breast imaging with PCCT offers an added advantage: it enables three-dimensional visualization of the breast tissue surrounding implants, aiding in the identification or the exclusion of suspicious breast lesions [12, 15]. Due to the high spatial resolution and the depiction of contrast uptake, PCCT might additionally be expected to show convincing performance in breast lesion characterization similar to dual-energy CT [23], dedicated breast CT [24], or breast MRI [18]. Also, a simultaneous assessment of axillary and internal mammary lymph nodes is provided with this method. Therefore, in addition to silicone breast implants, the whole breast might be evaluated with thoracic PCCT in the future, a perspective deserving further investigations.

The main limitations of our study certainly are the single-center design and a small sample size, which may restrict the generalizability of our findings. In addition, this small sample size does not include all degenerative changes of breast implants, particularly extracapsular ruptures were not represented. Finally, we performed thoracic PCCT in the prone position, which is not the standard of care; this means that the diagnostic performance we reported should be verified using the supine position when the examination will not be performed with the specific aim of breast implant evaluation. Aside, PCCT devices are not yet available on a large scale.

In conclusion, the results of our pilot study showed that silicone breast implants exhibit distinct contrast properties in thoracic PCCT, and that thoracic PCCT is highly accurate in detecting various relevant signs of degenerative changes and rupture of silicone breast implants. Therefore, in addition to dual-energy CT as shown in previous studies [12], thoracic PCCT shows promising results in the assessment of silicone breast implants and the diagnosis of implant integrity without the need of dedicated breast CT systems or more expensive and time-consuming breast MRI.

## Data Availability

The datasets used and/or analyzed during the current study are available from the corresponding author on reasonable request.

## Abbreviations

CI: Confidence interval
CNR: Contrast-to-noise ratio
CT: Computed tomography
MRI: Magnetic resonance imaging
PCCT: Photon-counting computed tomography
